# Difference in Computed Tomography Image Quality between Central Vein and Peripheral Vein Enhancement in Treatment Naive Esophageal Cancer Patients

**DOI:** 10.3390/cancers13164172

**Published:** 2021-08-19

**Authors:** Chun-Bi Chang, Chien-Cheng Chen, Huan-Wu Chen, Ching-Feng Wu, Jui-Ying Fu, Ming-Ju Hsieh, Yang-Teng Peng, Ssu-Ying Lu, Ching-Yang Wu

**Affiliations:** 1 Department of Medical Imaging and Intervention, Chang Gung Memorial Hospital, Linkou 333423, Taiwan; cooler@cgmh.org.tw (C.-B.C.); chenchiencheng@gmail.com (C.-C.C.); b2401003@gmail.com (H.-W.C.); 2 Department of Medical Imaging and Radiological Sciences, College of Medicine, Chang Gung University, Taoyuan 33323, Taiwan; 3 Department of Medicine, Medical College, Chang Gung University, Taoyuan 33323, Taiwan; maple.bt88@gmail.com (C.-F.W.); juiing0917@hotmail.com (J.-Y.F.); hsiehmj2@cgmh.org.tw (M.-J.H.); 4 Division of Thoracic and Cardiovascular Surgery, Department of Surgery, Chang Gung Memorial Hospital, Linkou 333423, Taiwan; ying772677@gmail.com; 5 Department of Internal Medicine, Division of Pulmonary and Critical Care Medicine, Chang Gung Memorial Hospital, Linkou 333423, Taiwan; 6 Institute of Epidemiology and Preventive Medicine, College of Public Health, National Taiwan University, Taipei 100025, Taiwan; k74866740@gmail.com

**Keywords:** computed tomography, esophageal cancer, central vein enhancement

## Abstract

**Simple Summary:**

A chest CT via central vein enhancement not only eliminates peripheral vein regurgitation but also provides better image quality that facilitates precise clinical staging. A chest CT via central vein enhancement may be considered after tissue proof in order to better discriminate disease severity.

**Abstract:**

The differences in chest computed tomography (CT) image quality may affect the tumor stage. The aim of this study was to compare the image quality and accuracy of chest CT via central vein and peripheral vein enhancement. Fifty consecutive patients were enrolled from a tertiary medical center in Taiwan from May 2016 to March 2019. All the patients received a chest CT via central vein enhancement prior to neoadjuvant concurrent chemoradiation in order to compare the chest CT that was obtained via the peripheral vein. In addition, blind independent central reviews of chest CT via central vein and peripheral vein enhancement were conducted. For T and N stage, chest CT via central vein enhancement had a greater consistency with endoscopic ultrasonography and positron-emission tomography-computed tomography findings (kappa coefficients 0.4471 and 0.5564, respectively). In addition, chest CT via central vein enhancement also showed excellent agreement in the blind independent central review (kappa coefficient 0.9157). The changes in the T and N stage resulted in stage migration in 16 patients. Chest CT via central vein enhancement eliminated peripheral vein regurgitation and also provided more precise clinical staging. This study is registered under the registered NCT number 02887261.

## 1. Introduction

A secure vascular access is crucial for oncology patients, such as those with esophageal cancer [1]. Various medications including chemotherapy and parenteral nutrition need to be given via a peripheral vein; however, these medications are highly irritant to the endothelium and can result in venous thrombosis. Consequently, repeat venipunctures were needed until the central venous puncture technique was developed by Aubaniac in 1952 [2]. Even with this technique, it was still necessary to regularly replace the central venous catheter until Broviac developed a new catheter in 1973 [3]. Hickman further modified the design and added a subcutaneous cuff that could completely separate the proximal catheter and extracorporeal injection part [4]. The first totally implantable venous device, i.e., an intravenous port, was designed by Niederhuber and introduced into clinical practice in 1982 [5]. However, the pressure rating of an intravenous port is 10 to 15 atm (145 to 174 psi), which is not suitable for the injection of contrast medium [6]. Therefore, the need for venous puncture cannot be completely avoided, and the possibility of extravasation remains. Furthermore, skin and soft tissue necrosis can arise if contrast extravasation occurs [7,8]. In order to overcome this problem, a power injectable port that is rated for high pressure injections (300 to 325 psi) was designed and is widely utilized in clinical practice.

**Figure 1:**
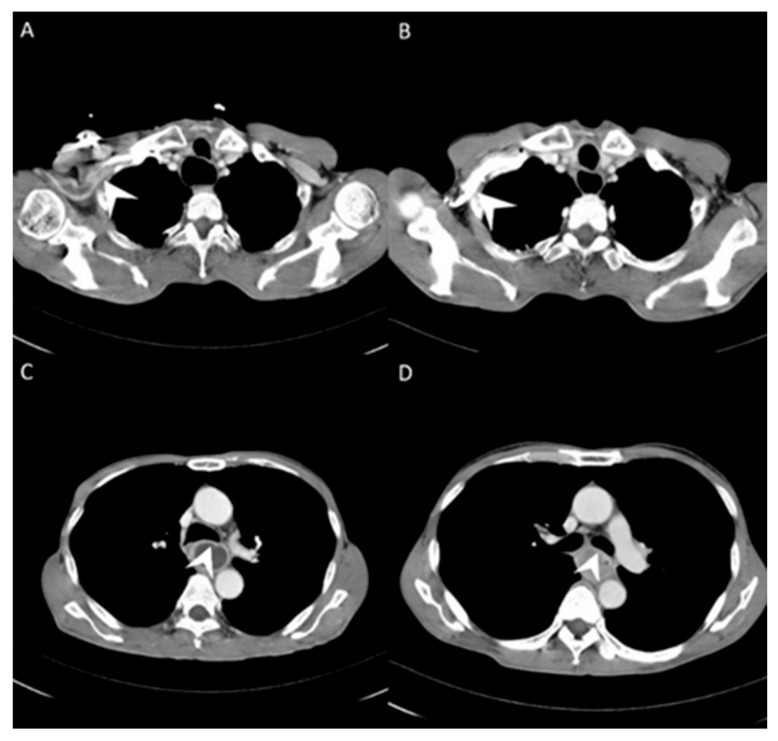
(**A**) Less contrast pooling within the subclavian vein (white arrow) was revealed with contrast injected via the power port (central vein). (**B**) More contrast pooling within the subclavian vein (white arrow) was identified with contrast injected via the peripheral vein. (**C**) Clear tumor margin (white arrow) was identified with contrast injected via the power port (central vein). (**D**) Blurred tumor margin (white arrow) with contrast injected via the peripheral vein.

For esophageal cancer patients, disease severity depends on serial imaging surveys including endoscopic ultrasonography (EUS), computed tomography (CT), and positron-emission tomography (PET) [9,10,11,12,13,14,15,16,17,18]. The patients who receive complete tumor resection have a better survival than those with inoperable tumors. Therefore, imaging surveys for tumor invasion to vital structures such as the trachea and descending aorta are crucial to evaluate resectability. All the imaging modalities have limitations that can lead to misinterpretation. EUS is an experience-dependent modality, and fibrotic changes caused by neoadjuvant therapy can make it difficult to evaluate invasion [17,18]. PET is a metabolic survey that can suggest possible distant metastatic lesions but not local tumor invasion. CT is used to clarify local invasiveness; however, the image quality varies according to when the scan is performed and the concentration of the intravascular contrast medium. In addition, CT uses spiral acquisitions that are reconstructed using computer software, and, thus, the image does not represent the actual disease status [19,20]. CT image quality has been correlated with injection flow [21]. A lower injection flow rate means that a longer period of time is required for the contrast medium to enter and become evenly distributed in the circulation, and five to seven whole-body circulations after completing the injection are needed before performing a CT scan. Accordingly, variations exist between the timing of performing a scan, and severe artifacts at the injection site can occur if the image is taken too early, and decreased sharpness can occur if the image is taken too late (Figure 1A,B). A higher injection flow rate means that more contrast medium can enter the circulation in a short time, resulting in a higher immediate concentration without the need to wait. This would then lead to consistency in the image timing and minimize variations in image quality. Theoretically, a central venous contrast medium injection could achieve a higher contrast concentration in a shorter time, and result in better image quality (Figure 1C,D). Moreover, better image quality would enhance the detection of small metastatic lesions and tumor invasion to the surrounding structures. The aim of this study was to analyze and compare the quality and accuracy of CT images obtained after the injection of contrast medium via a power injectable port and those obtained after the injection of contrast medium via a peripheral vein.

## 2. Materials and Methods

### 2.1. Patients and Enrollment

From May 2016 to March 2019, 325 esophageal cancer patients were evaluated by endoscopy, EUS, chest CT, and PET-CT, and classified according to the American Joint Commission on Cancer (AJCC) 8th TNM classification system [22,23]. One hundred of the patients staged as T3 to T4 NxM0 were recruited. All of these patients underwent prospective randomization and were assigned to power port and conventional groups (50 patients in each group). Only the patients who were assigned to the power port group received addition chest CT via central vein enhancement prior to neoadjuvant concurrent chemoradiation, and they were enrolled into this study in order to compare the chest CT image quality obtained using contrast injection via a peripheral vein and central vein (Figure S1). Patients who were assigned to the conventional group were excluded from this image comparison study. Image characteristics were compared among both series of images in the same patient prior to cancer treatment. All digital data and medical information were collected and recorded. This study was approved by the Institutional Review Board of Chang Gung Medical Foundation under the approval number 201503143A3. This study was supported by the Becton, Dickinson and Company, Franklin Lakes, NJ, USA; under the following grant numbers: XPRPG3F011, XPRPG3F012, XPRPG3F013, XPRPG3F014. The benefits and risks were explained to the patients, and informed consent forms were signed by the patients and their family. This study is registered at www.clinicaltrials.gov at 2 September 2016, under the registered NCT number 02887261.

### 2.2. Image Settings

#### 2.2.1. Chest CT via Central Vein Enhancement

Chest CT via central vein enhancement was performed using 64-slice CT (Toshiba Aquilion 32 CT machine, Toshiba America Medical Systems, Inc., Tustin, CA, USA). The image settings were 120 kV, 160 mA, 32 × 0.5 mm slices. Contrast medium (100 mL) (Omnipaque, Iohexol, GE Healthcare AS, Oslo, Norway) was injected using an injector (Medrad power injector, Bayer Medical Care Inc., Warrendale, PA, USA) at an injection rate of 3 mL/s via a Bard power port (Becton, Dickson and Company, Franklin Lakes, NJ, USA). The patient was placed in a feet first supine position, and the topogram direction was craniocaudal. The time of the first image was defined as when the CT number of the aortic arch reached 120 HU, and the time of the second image was defined as 20 s after the first image. The scan mode was helical, and the pitch and rotation time were 0.83 and 0.5 s, respectively. The scan was performed from the base of the skull to pubic symphysis, and the reconstruction slice thickness was 5 mm.

#### 2.2.2. Chest CT via Peripheral Vein Enhancement

The settings for chest CT via peripheral vein enhancement were the same as for chest CT via central vein enhancement, except that the injection rate was 1 mL/s.

### 2.3. Blind Independent Central Review 

Blind independent central review (BICR) was conducted according to the “Two Readers and Adjudicator Paradigm” [24,25,26]. Two independent radiologists re-evaluated the CT images via peripheral vein and central vein enhancement. All key variables were re-evaluated. If both radiologists agreed on the image reading, the assessment was complete. However, if the two radiologists could not reach a consensus, a third radiologist re-evaluated the CT images (Figure S2).

### 2.4. Statistical Analysis

All collected clinico-pathologic factors were evaluated using univariate analysis. Categorical variables were compared using the chi-square test and Fisher’s exact test, while continuous variables were compared using the two-sample *t*-test. Cohen’s kappa coefficients were used to compare differences between the different image staging tools and the radiologists’ readings. A *p*-value < 0.05 was considered to indicate a statistically significant difference. All analyses were performed using SAS, version 9 (SAS Institute, Cary, NC, USA).

## 3. Results

### 3.1. Image Differences between Central Vein Enhanced and Peripheral Vein Enhanced CT

CT number, i.e., Hounsfield scale, is a quantitative scale used to describe radiodensity. Table 1 shows the descriptive data of the patients, the CT number of the background, and the tumor severity and tumor measurements. The radiodensity between CT via peripheral vein and central vein enhancement was different except for the liver. Under the same timing of the imaging, i.e., the CT number of the aortic arch reached 120 HU, the radiodensity of all the tumor characteristics were similar between the CT via central vein and peripheral vein enhancement except for peripheral vein regurgitation (Figure S3). The red arrow in Figure S3A indicates contrast medium pooling at the junction site of the left subclavian and internal jugular vein, and the red star in Figure S2B indicates contrast medium pooling at the entry site where the left subclavian vein enters the thoracic cage. No peripheral vein regurgitation was noted in the CT via central vein enhancement (Figure S3C,D). There were no significant differences in the tumor measurements, including tumor volume and esophageal wall thickness, between the CT via central vein and peripheral vein enhancement (Table 1).

### 3.2. Tumor Invasion and Lymph Node Extension in Central Vein and Peripheral Vein Enhanced CT

#### 3.2.1. Image Difference in Tumor Invasion Status

In order to compare the image quality of the CT via central vein and peripheral vein enhancement, the two sets of images were compared with regard to tumor invasion and lymph node extension status. In the T stage patients, we used EUS as the comparison reference because this image modality provides real-time imaging of tumor invasion status. There was fair agreement between the CT via peripheral vein enhancement and EUS (Table 2A and Table S1; kappa coefficient = 0.3620) but moderate agreement between the CT via central vein enhancement and EUS (Table 2B and Table S1; kappa coefficient = 0.4471). In addition, there was only slight agreement between the initial reading and the BICR of the CT via peripheral vein enhancement (Table 3A and Table S2; kappa coefficient = 0.2382). There was high agreement between the initial image reading and the BICR of the CT via central vein enhancement (Table 3B and Table S2, kappa coefficient = 0.9157). We further compared the agreement between the initial reading of the CT via peripheral vein enhancement and the BICR of the CT via central vein enhancement. We found only fair agreement in the T stage between the initial reading of the CT via peripheral vein enhancement and the BICR of CT via central vein enhancement (Table 3C and Table S2, kappa coefficient = 0.3755). Fourteen patients had a T stage revision and the reasons for revision and actual image comparisons are summarized in Figure S4. Five patients had airway invasion, in which the CT via central vein enhancement showed tissue boundary loss and small soft tissue protruding with the same radiodensity (Figure S4A,B). Four patients had a clearer tumor infiltration margin (Figure S4C,D), and three patients had a clearer aorta fat plane (Figure S4E,F). In addition, the central vein CT images also provided details of locally invasive lesions. One patient had a thin continuous flat plane even though the lesion encompassed a quarter of the circumference of the descending aorta (Figure S4G,H), and another patient had tumor ingrowth into the right atrium (Figure S4I,J).

#### 3.2.2. Image Difference in Lymph Node Extension

With regard to lymph node extension, i.e., N stage, we used a PET-CT as the comparison reference because it can reveal all lesions with an increased glucose metabolic activity that are suspected tumor metastases. We found that the CT via peripheral vein enhancement had fair agreement with the PET-CT (Table 4A and Table S3; kappa coefficient = 0.3986), but there was only moderate agreement between the CT via central vein enhancement and the PET-CT (Table 4B and Table S3; kappa coefficient = 0.4299). We also compared the agreement between the initial reading and the BICR of the CT via peripheral and central vein enhancement. There was moderate agreement between the initial reading and the BICR of the CT via peripheral vein enhancement (Table 5A and Table S4; kappa coefficient = 0.5565). However, there was almost perfect agreement between the initial image reading and the BICR of CT via central vein enhancement (Table 5B and Table S4, kappa coefficient = 0.9425). There was moderate agreement between the initial reading results of the CT via peripheral vein enhancement and the BICR of CT via central vein enhancement, which was similar to the BICR of the CT via peripheral vein enhancement (Table 5C and Table S4; kappa coefficient = 0.5951). Twelve patients had an N stage revision, and there were three major reasons for the revisions. Six patients had small lymph nodes and the clinical stage was downgraded (Figure S5). Four patients were ungraded because of a necrotic node or a large or a cluster of lymph nodes (Figure S5). Two patients were identified to have lymph nodes close to the tumor in the CT via central vein enhancement. In addition, the changes in the T and N stage resulted in stage migration in 16 patients of the study cohort (16/50, 32%, Table S5).

## 4. Discussion

CT images are grayscale images that are correlated with tissue density and show radiodensity. In this study, the radiodensity between the CT via peripheral vein and central vein enhancement was different except for the liver. The blood supply to the liver is quite different from other solid organs. The double blood supply, including the hepatic artery and portal vein, may result in contrast pooling within parenchyma and reveal similar radiodensity during image acquisition. With regard to esophageal tumors, the CT via peripheral and central vein enhancement showed similar radiodensity in this study. Blood supply to the esophagus is via small vessels that originate from the aorta and are difficult to enhance. Accordingly, the radiodensity of esophageal tumors presents as soft tissue without variations in enhancement. In our study, similar esophageal tumor volume (*p* = 0.9990) and wall thickness (*p* = 0.9185) were identified in the CT via central and peripheral vein enhancement. In addition, peripheral vein regurgitation was completely eliminated in the CT via central vein enhancement (*p* = 0.0008, Figure S3), thereby avoiding artifacts that could interfere with discriminating between lymph nodes located in the superior vena cava and the surrounding soft tissue. In addition, the implanted catheter did not show any artifacts in the non-contrast CT and during imaging after power injection, i.e., central vein enhanced CT (Figure S6). Furthermore, the radiodensity of the ascending aorta (*p* = 0.0009) and descending aorta (*p* = 0.0012) of the CT via peripheral vein enhancement showed a higher variation than the CT via central vein enhancement. This was due to variations in intravascular contrast concentration during image acquisition leading to a possible over-enhancement or under-enhancement of the surrounding background. The former would lead to bright enhancement around the aorta, and the latter would lead to decreased aorta enhancement. Therefore, the CT via central vein enhancement showed a constant background that could provide more detailed information and facilitate interpretation by radiologists. With regard to the T stage, EUS has been shown to be more accurate than CT for the evaluation of tumor invasion status [11,27,28,29,30,31]. Only fair agreement between the CT via peripheral vein enhancement and EUS was found in this study (kappa coefficient = 0.3620). The correlation between the central vein enhanced CT and EUS showed moderate agreement (kappa coefficient = 0.4417). This finding may imply better tumor invasive status with the CT via central vein enhancement. In addition, reproducibility is also crucial, and we further investigated the agreement between the initial reading and the BICR. In the CT via central vein enhancement, there was high agreement between the initial reading and the BICR (kappa coefficient = 0.9157). However, only slight agreement between the initial reading and the BICR was identified in the CT via peripheral vein enhancement (kappa coefficient = 0.2382). Taken together, these findings showed the high reproducibility of T stage with the CT via central vein enhancement. We further compared the results of the initial reading of the CT via peripheral vein enhancement and the BICR of the CT via central vein enhancement, and only fair agreement was identified (kappa coefficient = 0.3755). With regard to the images, the CT via central vein enhancement had more clear tumor infiltrates and borders (Figure S4). These findings imply that the CT via central vein enhancement was more accurate than the CT via peripheral vein enhancement in detecting the tumor invasion status.

With regard to the N stage, CT and PET-CT were used to evaluate lymph node involvement. PET-CT is a physiological examination that reveals glucose metabolic activity, whereas CT reveals the penetration status of radiation and is correlated with radiodensity. In comparisons of the agreement between CT and PET-CT, we found that the CT via central vein enhancement (kappa coefficient = 0.4299) had better agreement with PET-CT than the CT via peripheral vein enhancement (kappa coefficient = 0.3986). With regard to reproducibility, we re-analyzed the agreement between the initial reading and the BICR. Only moderate agreement was found between the initial reading and the BICR in the CT via peripheral vein enhancement (kappa coefficient = 0.5565); however, there was almost perfect agreement between the initial image reading and the BICR in the CT via central vein enhancement (kappa coefficient = 0.9425). In addition, the correlation between the CT via peripheral vein enhancement and the BICR of the CT via central vein enhancement (kappa coefficient = 0.5951). This finding clarified that the CT via peripheral vein enhancement was less able to identify lymph node involvement and also had worse reproducibility. This may have been correlated to variations in the background, which may have blurred the border of suspected lymph nodes.

The power injectable port and lock were crucial for power injection. The power injectable port had a relative rigid and over-sized silicone diaphragm in order to minimize the change of shape during high pressure injection and provided strong bite force to keep the non-coring needle in situ. The power injectable lock had structural strength in the extension line of the non-coring needle. From the literature reviews, preliminary results showed the power port to have a similar complication rate as conventional ports [32,33]. As the power port could serve as entry access for the chemotherapeutic agent and contrast medium, the frequency of venipuncture was much decreased, and the patients’ satisfaction were higher than conventional port [34]. In our study, there was no contrast leak during power injection and may be correlated to the low body mass index of our study cohort. A further safety investigation of the power injection was warranted.

There are several limitations to this study. The number of cases was small, and, thus, subgrouping of patients according to tumor stage was not possible. In addition, the tumor invasion status of this cohort was T3 to T4. Further investigations are warranted to clarify the difference in image quality between CT via central vein enhancement and via peripheral vein enhancement in patients with T1 or T2 status. Despite these limitations, the CT via central vein enhancement had better image quality, that not only minimized misinterpretation but also provided precise tumor staging. Our findings may assist in pre-treatment planning for esophageal cancer patients who present as T3-T4 Nx M0.

## 5. Conclusions

Central vein CT not only eliminated peripheral vein regurgitation but also had less background variation. Both characteristics resulted in high reproducibility among radiologists and stage migration in 32% of T3-4 NxM0 esophageal cancer patients.

## Figures and Tables

**Table 1:** Patient and image characteristics of the study group.

Patient Characteristics	Value (Mean ± SD)		
Number of patients	48		
Sex	Female = 2/Male = 46		
Age (years)	57.94 ± 8.61		
Image characteristics	Value (mean ± SD)		
	CT (via the peripheral vein)	CT (via the central vein)	*p* Value
Tumor measurement			
Pre-Tx CT tumor volume	46.43 ± 39.64	46.44 ± 39.89	0.999
Pre-Tx wall thickness	16.48 ± 5.79	16.36 ± 5.75	0.9185
Pre Tx LN station	3.50 ± 2.73	3.50 ± 2.73	1
CT number of major structures			
Pre-Tx ascending aorta	189.71 ± 41.43	164.97 ± 27.51	0.0009
Pre-Tx pulmonary artery	173.43 ± 42.68	156.40 ± 25.07	0.0197
Pre-Tx descending aorta	186.29 ± 39.80	163.28 ± 26.12	0.0012
Pre-Tx liver	105.10 ± 17.47	115.90 ± 15.30	1
Pre-Tx kidney	173.96 ± 27.81	190.76 ± 30.59	0.0059
CT measurement of tumor			
Pre-Tx tumor	77.30 ± 13.4	79.45 ± 13.12	0.4281
Pre-Tx LN	78.54 ± 20.83	68.65 ± 35.54	0.1065
Pre-Tx peripheral vein regurgitation	N = 38/Y = 10	N = 48	0.0008

**Table 2:** T stage agreement between CT enhancement via peripheral vein and central vein compared with EUS.

(**A**) Agreement of T stage (peripheral vein CT vs. EUS)								
**Coincidence of T Stage: Peripheral Vein CT (p-CT) versus Endoscopic Sonography (EUS)**								
	EUS	T stage					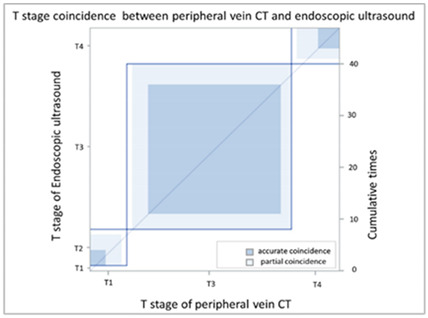	
p-CT		T1	T2	T3	T4	Total		
T stage	T1	**0**	0	0	0	0		
	T2	0	**3**	3	1	7		
	T3	1	3	**25**	2	31		
	T4	0	1	4	**4**	9		
	Total	1	7	32	7	**47**		
Kappa coefficient				Adjust standard error				95% confidential interval
0.3620				0.1236				0.1196-0.6403
(**B**) Agreement of T stage (central vein CT vs. EUS)								
**Coincidence of T Stage: Central Vein CT (c-CT) versus Endoscopic Sonography (EUS)**								
	EUS	T stage					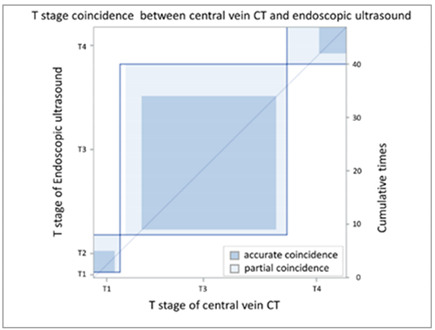	
p-CT		T1	T2	T3	T4	Total		
T stage	T1	**0**	0	0	0	0		
	T2	0	**4**	1	0	5		
	T3	1	3	**25**	2	31		
	T4	0	0	6	**5**	11		
	Total	1	7	32	7	**47**		
Kappa coefficient				Adjust standard error				95% confidential interval
0.4471				0.1275				0.1972-0.6969

**Table 3:** T stage agreement between CT enhancement via peripheral vein CT and central vein (blind independent central review).

(**A**) Agreement between T stage of initial reading (peripheral vein CT) versus BICR (peripheral vein CT)								
**T Stage Agreement: Reading Result of Peripheral Vein CT (p-CT): Initial Reading versus BICR**								
	Initial p-CT	T stage					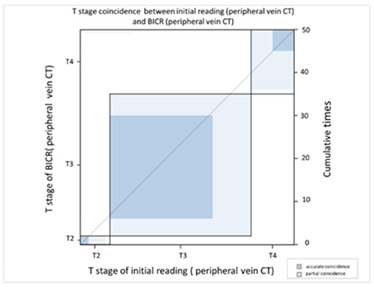	
BICR p-CT		T1	T2	T3	T4	Total		
T stage	T1	**0**	0	0	0	0		
	T2	0	**2**	4	1	7		
	T3	0	0	**24**	9	33		
	T4	0	0	5	**5**	10		
	Total	0	2	35	15	**50**		
Kappa coefficient				Adjust standard error				95% confidential interval
0.2382				0.1273				0.0114-0.4788
(**B**) Agreement between T stage initial reading (central vein CT) versus BICR (central vein CT)								
**T Stage Agreement: Reading Result of Central Vein CT (c-CT): Initial Reading versus BICR**								
	Initial p-CT	T stage					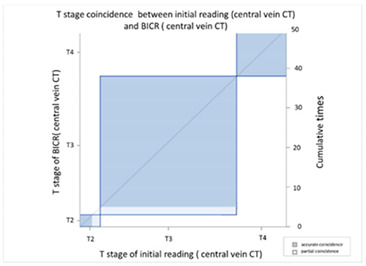	
BICR p-CT		T1	T2	T3	T4	Total		
T stage	T1	**0**	0	0	0	0		
	T2	0	**3**	2	0	5		
	T3	0	0	**33**	0	33		
	T4	0	0	0	**12**	12		
	Total	0	3	35	12	**50**		
Kappa coefficient				Adjust standard error				95% confidential interval
0.9157				0.0578				0.8025-1.0000
(**C**) Agreement between T stage initial reading (peripheral vein CT) versus BICR (central vein CT)								
**T Stage Agreement: Initial Reading of Peripheral Vein CT (p-CT) versus BICR of Central Vein CT (c-CT)**								
	Initial p-CT	T stage					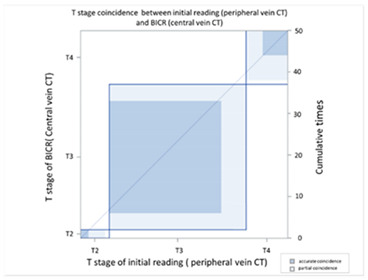	
BICR p-CT		T1	T2	T3	T4	Total		
T stage	T1	**0**	0	0	0	0		
	T2	0	**2**	4	1	7		
	T3	0	0	**27**	6	33		
	T4	0	0	4	**6**	10		
	Total	0	2	35	13	**50**		
Kappa coefficient				Adjust standard error				95% confidential interval
0.3755				0.1257				0.1293-0.6218

**Table 4:** N stage agreement between CT enhancement via peripheral vein and central vein compared with PET.

(**A**) Agreement of N stage (peripheral vein CT vs. PET)								
**N Stage Agreement: Initial Reading of Peripheral Vein CT (p-CT) versus Positron Emission Tomography (PET)**								
	p-CT	N stage					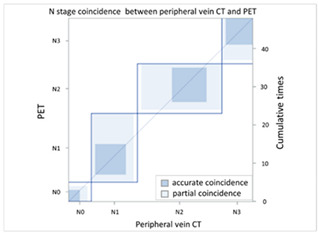	
PET		N0	N1	N2	N3	Total		
N stage	N0	**3**	2	1	0	6		
	N1	1	**8**	2	1	12		
	N2	1	8	**9**	4	22		
	N3	0	0	1	**7**	8		
	Total	5	18	13	12	**48**		
Kappa coefficient				Adjust standard error				95% confidential interval
0.3986				0.0979				0.2068-0.5904
(**B**) Agreement of N stage (central vein CT vs. PET)								
**N Stage Agreement: Initial Reading of Peripheral Vein CT (p-CT) versus Positron Emission Tomography (PET)**								
	p-CT	N stage					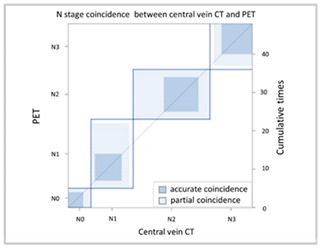	
PET		N0	N1	N2	N3	Total		
N stage	N0	**4**	2	0	0	6		
	N1	1	**7**	2	1	11		
	N2	0	8	**9**	3	20		
	N3	0	1	2	**8**	11		
	Total	5	18	13	12	**48**		
Kappa coefficient				Adjust standard error				95% confidential interval
0.4299				0.0981				0.2367-0.6223

**Table 5:** N stage agreement between CT enhancement via peripheral vein and central vein (blind independent central review).

(A) Agreement between N stage of the initial reading (peripheral vein CT) versus BICR (peripheral vein CT)								
**N Stage Agreement of Peripheral Vein CT (p-CT): Initial Reading versus BICR**								
	p-CT	N stage					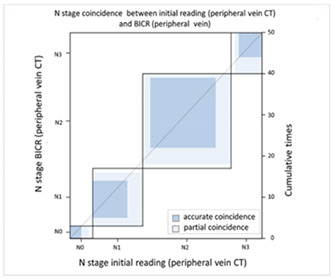	
PET		N0	N1	N2	N3	Total		
N stage	N0	**3**	2	1	0	6		
	N1	0	**9**	4	0	13		
	N2	0	2	**17**	4	23		
	N3	0	1	1	**6**	8		
	Total	3	14	23	10	**50**		
Kappa coefficient				Adjust standard error				95% confidential interval
0.5565				0.0961				0.3681-0.7499
(**B**) Agreement between N stage of the initial reading (central vein CT) versus BICR (central vein CT)								
**N Stage Agreement of Central Vein CT (c-CT): Initial Reading versus BICR**								
	p-CT	N stage					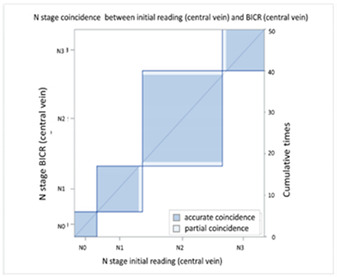	
PET		N0	N1	N2	N3	Total		
N stage	N0	**6**	0	0	0	6		
	N1	0	**11**	1	0	12		
	N2	0	0	**21**	0	21		
	N3	0	0	1	**10**	11		
	Total	6	11	23	10	**50**		
Kappa coefficient				Adjust standard error				95% confidential interval
0.9425				0.04				0.8641-1.0000
(**C**) Agreement between N stage of the initial reading (peripheral vein CT) versus BICR (central vein CT)								
**N Stage Agreement of Central Vein CT (c-CT): Initial Reading versus BICR**								
	p-CT	N stage					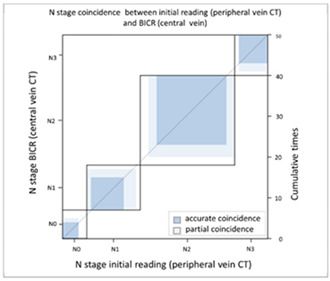	
PET		N0	N1	N2	N3	Total		
N stage	N0	**4**	0	2	0	6		
	N1	1	**8**	3	1	13		
	N2	2	2	**17**	2	23		
	N3	0	1	0	**7**	8		
	Total	7	11	22	10	**50**		
Kappa coefficient				Adjust standard error				95% confidential interval
0.5951				0.092				0.4149–0.7754

## Data Availability

Data available on request due to restrictions. The data presented in this study are available on request from the corresponding author. The data are not publicly available due to regulation of personal information protection.

## Supplementary Materials

The following are available online at https://www.mdpi.com/article/10.3390/cancers13164172/s1, Figure S1. Study algorithm, Figure S2. Blind independent image review, Figure S3. Peripheral vein regurgitation, Figure S4. Differences in T stage between peripheral vein and central vein CT and the reasons for T stage revision, Figure S5. Differences in N stage between peripheral vein and central vein CT and the reasons for N stage revision, Figure S6 Actual catheter image in non-enhanced and central vein enhanced CT, Table S1. Detailed status of T stage among different imaging tools in the power port group (peripheral vein CT versus central vein CT versus EUS), Table S2. Details of T stage in initial and revised reading between peripheral vein and central vein CT, Table S3. Detailed status of N stage among different imaging tools in the power port group (peripheral vein CT versus central vein CT versus PET), Table S4. Details of revised N stage in blind independent radiologist review of peripheral vein CT and central vein CT, Table S5. Stage migration after stage revision by central vein CT (pre-revision).

## References

[1] Charvát J., Linke Z., Horáèková M., Prausová J. (2006). Implantation of central venous ports with catheter nsertion via the right internal jugular vein in oncology patients: Single center experience. Support. Care Cancer.

[2] Copper C.M., Pacanowski J.P., Bell J.L. (2005). The trapezius port: A novel approach for port access. Am. Surg..

[3] Broviac J.W., Cole J.J., Scribner B.H. (1973). A silicone rubber atrial catheter for prolonged parenteral alimentation. Surg. Gynecol. Obstet..

[4] Hickman R.O., Buckner C.D., Clift R.A., Sanders J.E., Stewart P., Thomas E.D. (1979). A modified right atrial catheter for access to the venous system in marrow transplant recipients. Surg. Gynecol. Obstet..

[5] Niederhuber J.E., Ensminger W., Gyves J.W., Liepman M., Doan K., Cozzi E. (1982). Totally implanted venous and arterial access system to replace external catheters in cancer treatment. Surgery.

[6] Teichgräber U.K., Gebauer B., Benter T., Wagner H.J. (2003). Central venous access catheters: Radiological management of complications. Cardiovasc. Interv. Radiol..

[7] Belzunegui T., Louis C.J., Torrededia L., Oteiza J. (2011). Extravasation of radiographic contrast material and compartment syndrome in the hand: A case report. Scand. J. Trauma Resusc. Emerg. Med..

[8] Azaïs H., Bresson L., Bassil A., Katdare N., Merlot B., Houpeau J.L., El Bedoui S., Meurant J.P., Tresch E., Narducci F. (2014). Chemotherapy drug extravasation in totally implantable venous access port systems: How effective is early surgical lavage?. J. Vasc. Access.

[9] Rice T.W. (2000). Clinical staging of esophageal carcinoma. CT, EUS, and PET. Chest Surg. Clin. N. Am..

[10] Wani S., Das A., Rastogi A., Drahos J., Ricker W., Parsons R., Bansal A., Yen R., Hosford L., Jankowski M. (2015). Endoscopic ultrasonography in esophageal cancer leads to improved survival rates: Results from a population-based study. Cancer.

[11] Yen T.J., Chung C.S., Wu Y.W., Yen R.F., Cheng M.F., Lee J.M., Hsu C.H., Chang Y.L., Wang H.P. (2012). Comparative study between endoscopic ultrasonography and positron emission tomography-computed tomography in staging patients with esophageal squamous cell carcinoma. Dis. Esophagus.

[12] Cerfolio R.J., Bryant A.S., Ohja B., Bartolucci A.A., Eloubeidi M.A. (2005). The accuracy of endoscopic ultrasonography with fine-needle aspiration, integrated positron emission tomography with computed tomography, and computed tomography in restaging patients with esophageal cancer after neoadjuvant chemoradiotherapy. J. Thorac. Cardiovasc. Surg..

[13] Sandha G.S., Severin D., Postema E., McEwan A., Stewart K. (2008). Is positron emission tomography useful in locoregional staging of esophageal cancer? Results of a multidisciplinary initiative comparing CT, positron emission tomography, and EUS. Gastrointest. Endosc..

[14] Pfau P.R., Perlman S.B., Stanko P., Frick T.J., Gopal D.V., Said A., Zhang Z., Weigel T. (2007). The role and clinical value of EUS in a multimodality esophageal carcinoma staging program with CT and positron emission tomography. Gastrointest. Endosc..

[15] Sloof G.W. (2006). Response monitoring of neoadjuvant therapy using CT, EUS, and FDG-PET. Best Pract. Res. Clin. Gastroenterol..

[16] Yegin E.G., Duman D.G. (2014). Staging of esophageal and gastric cancer in 2014. Minerva Med..

[17] Isenberg G., Chak A., Canto M.I., Levitan N., Clayman J., Pollack B.J., Sivak M.V. (1998). Endoscopic ultrasound in restaging of esophageal cancer after neoadjuvant chemoradiation. Gastrointest. Endosc..

[18] Kalha I., Kaw M., Fukami N., Patel M., Singh S., Gagneja H., Cohen D., Morris J. (2004). The accuracy of endoscopic ultrasound for restaging esophageal carcinoma after chemoradiation therapy. Cancer.

[19] Van den Hoed R.D., Feldberg M.A., van Leeuwen M.S., van Dalen T., Obertop H., Kooyman C.D., van der Schouw Y.T., de Graaf P.W. (1997). CT prediction of irresectability in esophageal carcinoma: Value of additional patient positions and relation to patient outcome. Abdom. Imaging.

[20] Tsujimoto H., Ichikura T., Aiko S., Yaguchi Y., Kumano I., Takahata R., Matsumoto Y., Yoshida K., Ono S., Yamamoto J. (2012). Multidetector-computed tomography attenuation values between the tumor and aortic wall in response to induction therapy for esophageal cancer and its predictive value for aortic invasion. Exp. Ther. Med..

[21] Sun Y., Hua Y., Wang M., Mao D., Jin X., Li C., Shi K., Xu J. (2017). Evaluation of a High Concentrated Contrast Media Injection Protocol in Combination with Low Tube Current for Dose Reduction in Coronary Computed Tomography Angiography: A Randomized, Two-center Prospective Study. Acad. Radiol..

[22] Rice T.W., Ishwaran H., Hofstetter W.L., Kelsen D.P., Apperson-Hansen C., Blackstone E.H. (2016). Worldwide Esophageal Cancer Collaboration Investigators Recommendations for pathologic staging (pTNM) of cancer of the esophagus and esophagogastric junction for the 8th edition AJCC/UICC staging manuals. Dis. Esophagus.

[23] Rice T.W., Ishwaran H., Blackstone E.H., Hofstetter W.L., Kelsen D.P., Apperson-Hansen C., Worldwide Esophageal Cancer Collaboration Investigators (2016). Recommendations for clinical staging (cTNM) of cancer of the esophagus and esophagogastric junction for the 8th edition AJCC/UICC staging manuals. Dis. Esophagus.

[24] Cohen K.L., Gönen M., Ford R.R. (2015). Monitoring Reader Metrics in Blinded Independent Central Review of Oncology Studies. J. Clin. Trials.

[25] Amit O., Mannino F., Stone A.M., Bushnell W., Denne J., Helterbrand J., Burger H.U. (2011). Blinded independent central review of progression in cancer clinical trials: Results from a meta-analysis. Eur. J. Cancer.

[26] Dodd L.E., Korn E.L., Freidlin B., Jaffe C.C., Rubinstein L.V., Dancey J., Mooney M.M. (2008). Blinded independent central review of progression-free survival in phase III clinical trials: Important design element or unnecessary expense?. J. Clin. Oncol..

[27] Choi J., Kim S.G., Kim J.S., Jung H.C., Song I.S. (2010). Comparison of endoscopic ultrasonography (EUS), positron emission tomography (PET), and computed tomography (CT) in the preoperative locoregional staging of resectable esophageal cancer. Surg. Endosc..

[28] Luo L.N., He L.J., Gao X.Y., Huang X.X., Shan H.B., Luo G.Y., Li Y., Lin S.Y., Wang G.B., Zhang R. (2016). Endoscopic Ultrasound for Preoperative Esophageal Squamous Cell Carcinoma: A Meta-Analysis. PLoS ONE.

[29] Thosani N., Singh H., Kapadia A., Ochi N., Lee J.H., Ajani J., Swisher S.G., Hofstetter W.L., Guha S., Bhutani M.S. (2012). Diagnostic accuracy of EUS in differentiating mucosal versus submucosal invasion of superficial esophageal cancers: A systematic review and meta-analysis. Gastrointest. Endosc..

[30] Sun F., Chen T., Han J., Ye P., Hu J. (2015). Staging accuracy of endoscopic ultrasound for esophageal cancer after neoadjuvant chemotherapy: A meta-analysis and systematic review. Dis. Esophagus.

[31] Misra S., Choi M., Livingstone A.S., Franceschi D. (2012). The role of endoscopic ultrasound in assessing tumor response and staging after neoadjuvant chemotherapy for esophageal cancer. Surg. Endosc..

[32] Teichgräber U.K., Nagel S.N., Kausche S., Enzweiler C. (2012). Clinical benefit of power-injectable port systems: A prospective observational study. Eur. J. Radiol..

[33] Burbridge B., Plewes C., Stoneham G., Szkup P., Otani R., Babyn P., Bryce R. (2018). Randomized Clinical Trial Evaluating Complications and Complication-Related Removal of Arm-Situated Power-Injectable and Non-Power-Injectable Totally Implanted Venous Access Devices among Cancer Patients. J. Vasc. Interv. Radiol..

[34] Kunz-Virk J., Krüger K. (2019). Power-injectable totally implantable venous access devices-analysis of success and complication rates of ultrasound-guided implantation and a patient satisfaction. VASA.

