# Supplementary material for: Difference in Computed Tomography Image Quality between Central Vein and Peripheral Vein Enhancement in Treatment Naive Esophageal Cancer Patients
Source: Cancers (Basel). 2021 Aug 19;13(16):4172. doi: 10.3390/cancers13164172 (PMC8394425; doi:10.3390/cancers13164172)
Supplement: Supplementary file 1 [file cancers-13-04172-s001.zip › cancers-1324772-supplementary.pdf]

Index of the online-only supplements

Figure S1. Study algorithm.

Figure S2. Blind independent image review.

Figure S3. Peripheral vein regurgitation.

Table S1. Detailed status of T stage among different imaging tools in the power port group (peripheral vein CT versus central vein CT versus EUS).

Table S2. Details of T stage in initial and revised reading between peripheral vein and central vein CT.

Figure S4. Differences in T stage between peripheral vein and central vein CT and the reasons for T stage revision.

Table S3. Detailed status of N stage among different imaging tools in the power port group (peripheral vein CT versus central vein CT versus PET).

Table S4. Details of revised N stage in blind independent radiologist review of peripheral vein CT and central vein CT.

Figure S5. Differences in N stage between peripheral vein and central vein CT and the reasons for N stage revision.

Table S5. Stage migration after stage revision by central vein CT (pre-revision).

Figure S6 Actual catheter image in non-enhanced and central vein enhanced CT.

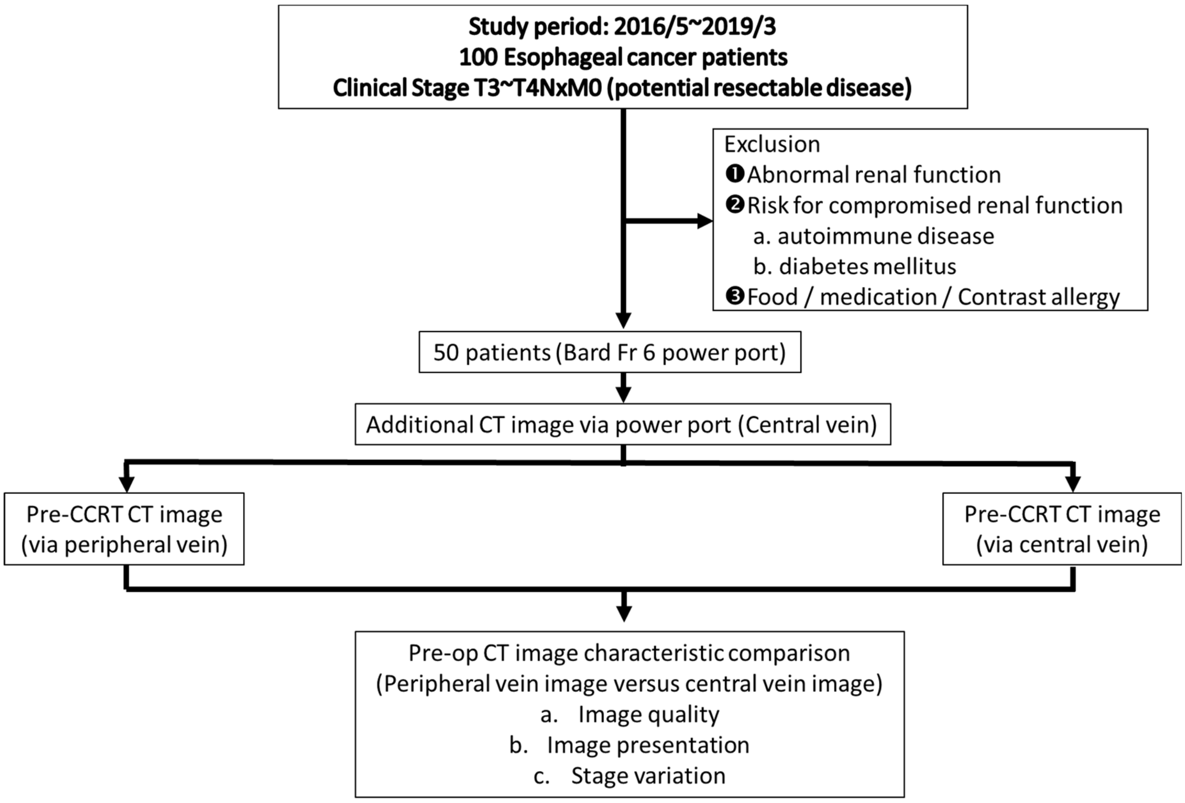

Figure S1. Study algorithm.

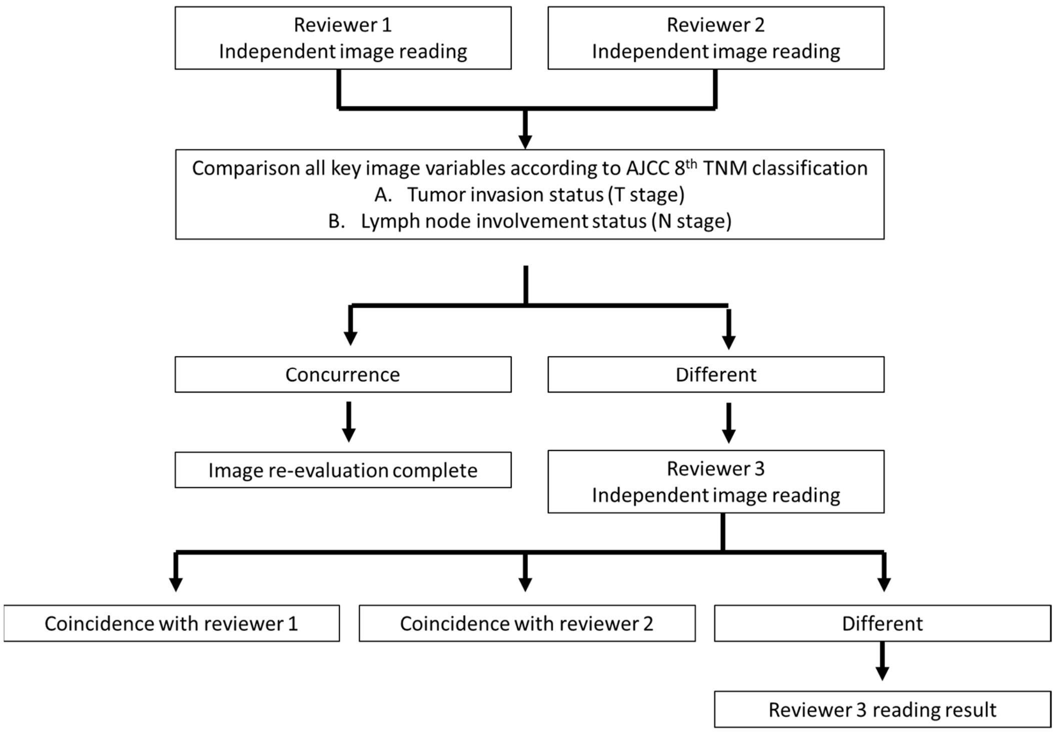

Figure S2. Blind independent image review.

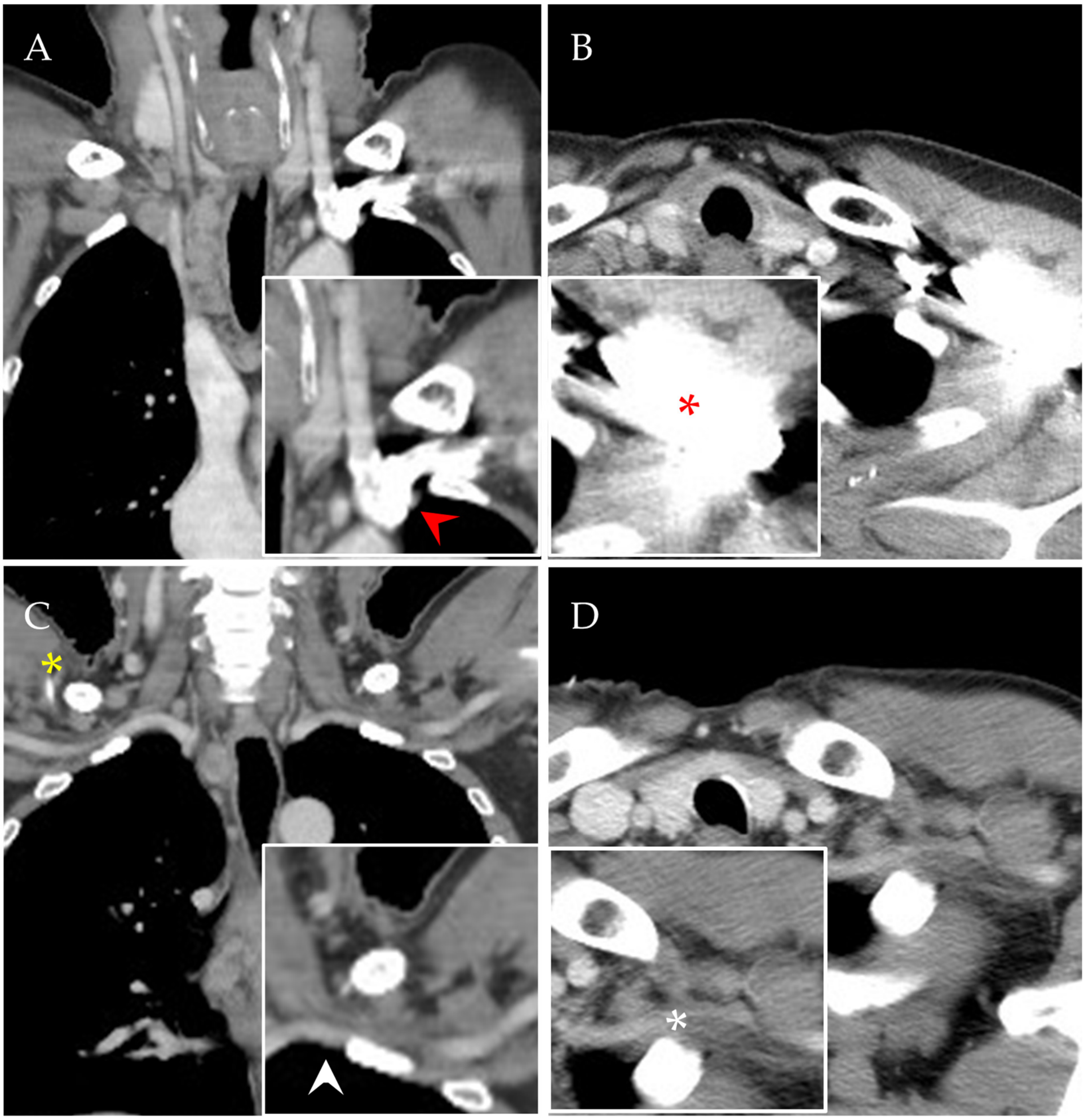

Figure S3. Peripheral vein regurgitation.

A. Peripheral vein regurgitation
   Contrast injected via left upper arm and pooling at junction site between left subclavian vein and left internal jugular vein. (red arrow, peripheral vein enhanced CT)
B. Cross section showed artifacts that caused by pooled contrast medium. (red star)
C. Contrast injected via power injectable port (yellow star) and no contrast medium pooling was noted (white arrow, central vein enhanced CT)
D. No contrast medium pooling and no more artifacts were noted (white star).

Table S1. Detailed status of T stage among different imaging tools in the power port group (peripheral vein CT versus central vein CT versus EUS)

Image / Patient	T stage		
	CT Image (peripheral vein)	CT Image (central vein)	Endoscopic ultrasound (EUS)
Case 1	T3	T3	T1
Case 2	T2	T2	T2
Case 3	T3	T3	T3
Case 4	T2	T2	T2
Case 5	T3	T3	T3
Case 6	T3	T3	T3
Case 7	T3	T2	T3
Case 8	T3	T4	T3
Case 9	T4	T3	T3
Case 10	T3	T3	T3
Case 11	T2	T2	T2
Case 12	T3	T3	T3
Case 13	T3	T3	T3
Case 14	T3	T3	T3
Case 15	T3	T3	T3
Case 16	T4	T3	T4
Case 17	T3	T3	T3
Case 18	T3	T3	T2
Case 19	T2	T3	T3
Case 20	T3	T3	T4
Case 21	T3	T3	T3
Case 22	T3	T3	T3
Case 23	T3	T3	T2
Case 24	T3	T3	T3
Case 25	T3	T3	T2
Case 26	T3	T3	T3
Case 27	T3	T4	T4
Case 28	T3	T3	Not done
Case 29	T3	T3	T3
Case 30	T4	T4	T4
Case 31	T4	T2	T2
Case 32	T3	T3	T3
Case 33	T4	T4	Not done
Case 34	T4	T4	T4
Case 35	T3	T3	T3
Case 36	T3	T3	T3
Case 37	T4	T4	T4
Case 38	T3	T3	T3
Case 39	T2	T3	T3
Case 40	T2	T3	T3
Case 41	T3	T3	T3
Case 42	T2	T4	T4
Case 43	T3	T3	Not done
Case 44	T3	T3	T3
Case 45	T3	T4	T3
Case 46	T4	T4	T3
Case 47	T3	T4	T3
Case 48	T4	T3	T3
Case 49	T4	T4	T3
Case 50	T3	T4	T3

Table S2. Details of T stage in initial and revised reading between peripheral vein and central vein CT

Image / Patient	T stage			
	CT Image (peripheral vein)	Revised CT image (peripheral vein)	CT Image (central vein)	Revised CT image (central vein)
Case 1	T3	T3	T3	T3
Case 2	T2	T3	T2	T2
Case 3	T3	T3	T3	T3
Case 4	T2	T2	T2	T2
Case 5	T3	T4	T3	T3
Case 6	T3	T4	T3	T3
Case 7	T3	T3	T2	T3
Case 8	T3	T4	T4	T4
Case 9	T4	T3	T3	T3
Case 10	T3	T3	T3	T3
Case 11	T2	T2	T2	T2
Case 12	T3	T3	T3	T3
Case 13	T3	T3	T3	T3
Case 14	T3	T3	T3	T3
Case 15	T3	T3	T3	T3
Case 16	T4	T3	T3	T3
Case 17	T3	T3	T3	T3
Case 18	T3	T3	T3	T3
Case 19	T2	T3	T3	T3
Case 20	T3	T3	T3	T3
Case 21	T3	T3	T3	T3
Case 22	T3	T3	T3	T3
Case 23	T3	T4	T3	T3
Case 24	T3	T4	T3	T3
Case 25	T3	T3	T3	T3
Case 26	T3	T3	T3	T3
Case 27	T3	T4	T4	T4
Case 28	T3	T3	T3	T3
Case 29	T3	T3	T3	T3
Case 30	T4	T4	T4	T4
Case 31	T4	T3	T2	T3
Case 32	T3	T3	T3	T3
Case 33	T4	T4	T4	T4
Case 34	T4	T3	T4	T4
Case 35	T3	T3	T3	T3
Case 36	T3	T3	T3	T3
Case 37	T4	T4	T4	T4
Case 38	T3	T3	T3	T3
Case 39	T2	T3	T3	T3
Case 40	T2	T3	T3	T3
Case 41	T3	T3	T3	T3
Case 42	T2	T4	T4	T4
Case 43	T3	T3	T3	T3
Case 44	T3	T3	T3	T3
Case 45	T3	T4	T4	T4
Case 46	T4	T4	T4	T4
Case 47	T3	T4	T4	T4
Case 48	T4	T3	T3	T3
Case 49	T4	T4	T4	T4
Case 50	T3	T4	T4	T4

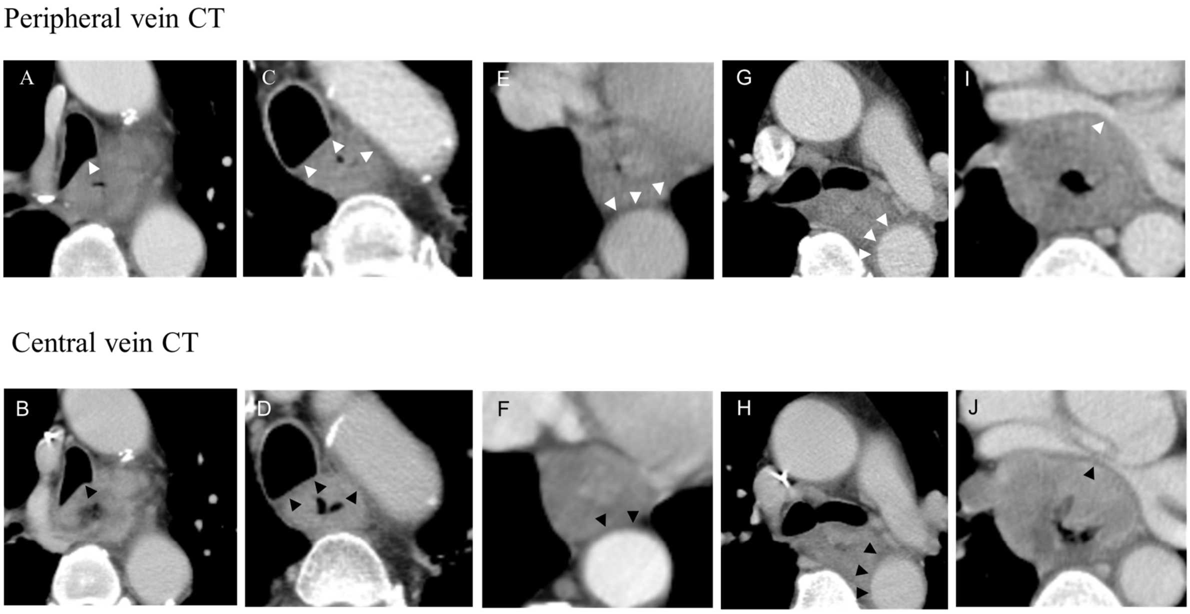

A (Peripheral vein CT)/ B (Central vein CT)	Airway invasion	Case number : 5
C (Peripheral vein CT)/ D (Central vein CT)	Tumor infiltrates	Case number : 4
E (Peripheral vein CT)/ F (Central vein CT)	Aorta clear fat plane	Case number : 3
G (Peripheral vein CT)/ H (Central vein CT)	Aorta invasion	Case number : 1
I (Peripheral vein CT)/ J (Central vein CT)	Right atrium invasion	Case number: 1

Figure S4. Differences in T stage between peripheral vein and central vein CT and the reasons for T stage revision.

Table S3. Detailed status of N stage among different imaging tools in the power port group (peripheral vein CT versus central vein CT versus PET)

Image / patient	N stage		
	CT Image (peripheral vein)	CT Image (central vein)	PET
Case 1	N2	N2	N2
Case 2	N3	N3	N3
Case 3	N2	N2	N2
Case 4	N2	N2	N2
Case 5	N2	N2	N1
Case 6	N3	N3	N3
Case 7	N3	N1	N3
Case 8	N2	N2	N3
Case 9	N2	N2	N1
Case 10	N0	N0	N1
Case 11	N1	N1	N2
Case 12	N2	N2	Not done
Case 13	N1	N3	N3
Case 14	N2	N2	N3
Case 15	N1	N1	N1
Case 16	N2	N2	N3
Case 17	N0	N0	N0
Case 18	N2	N1	N1
Case 19	N1	N2	N1
Case 20	N2	N0	N1
Case 21	N2	N2	N1
Case 22	N2	N2	N2
Case 23	N1	N3	N1
Case 24	N3	N3	N3
Case 25	N2	N3	N2
Case 26	N1	N1	N1
Case 27	N2	N2	N1
Case 28	N1	N1	N1
Case 29	N2	N2	N2
Case 30	N2	N3	N3
Case 31	N0	N0	N0
Case 32	N1	N1	N0
Case 33	N3	N3	N3
Case 34	N2	N2	N2
Case 35	N3	N3	N3
Case 36	N0	N1	N2
Case 37	N2	N0	N0
Case 38	N1	N1	N1
Case 39	N2	N2	N2
Case 40	N2	N2	N2
Case 41	N0	N0	N0
Case 42	N3	N3	N2
Case 43	N2	N1	N1
Case 44	N0	N2	N1
Case 45	N3	N3	N3
Case 46	N1	N1	N1
Case 47	N1	N2	N2
Case 48	N2	N2	N1
Case 49	N1	N1	Not done
Case 50	N1	N2	N1

Table S4. Details of revised N stage in blind independent radiologist review of peripheral vein CT and central vein CT

Image / Patient	N stage			
	CT Image (peripheral vein)	Revised CT image (peripheral vein)	CT Image (central vein)	Revised CT image (central vein)
Case 1	N2	N2	N2	N2
Case 2	N3	N3	N3	N3
Case 3	N2	N3	N2	N2
Case 4	N2	N2	N2	N2
Case 5	N2	N2	N2	N2
Case 6	N3	N2	N3	N3
Case 7	N3	N1	N1	N1
Case 8	N2	N2	N2	N2
Case 9	N2	N2	N2	N2
Case 10	N0	N0	N0	N0
Case 11	N1	N1	N1	N1
Case 12	N2	N2	N2	N2
Case 13	N1	N2	N3	N3
Case 14	N2	N3	N2	N2
Case 15	N1	N1	N1	N1
Case 16	N2	N2	N2	N2
Case 17	N0	N0	N0	N0
Case 18	N2	N1	N1	N1
Case 19	N1	N2	N2	N2
Case 20	N2	N2	N0	N0
Case 21	N2	N2	N2	N2
Case 22	N2	N2	N2	N2
Case 23	N1	N1	N3	N2
Case 24	N3	N3	N3	N3
Case 25	N2	N3	N3	N3
Case 26	N1	N1	N1	N1
Case 27	N2	N2	N2	N2
Case 28	N1	N1	N1	N1
Case 29	N2	N2	N2	N2
Case 30	N2	N3	N3	N3
Case 31	N0	N1	N0	N0
Case 32	N1	N1	N1	N1
Case 33	N3	N3	N3	N3
Case 34	N2	N2	N2	N2
Case 35	N3	N3	N3	N3
Case 36	N0	N1	N1	N2
Case 37	N2	N2	N0	N0
Case 38	N1	N1	N1	N1
Case 39	N2	N2	N2	N2
Case 40	N2	N2	N2	N2
Case 41	N0	N0	N0	N0
Case 42	N3	N3	N3	N3
Case 43	N2	N1	N1	N1
Case 44	N0	N2	N2	N2
Case 45	N3	N3	N3	N3
Case 46	N1	N1	N1	N1
Case 47	N1	N2	N2	N2

Case 48	N2	N2	N2	N2
Case 49	N1	N1	N1	N1
Case 50	N1	N2	N2	N2

Peripheral vein CT

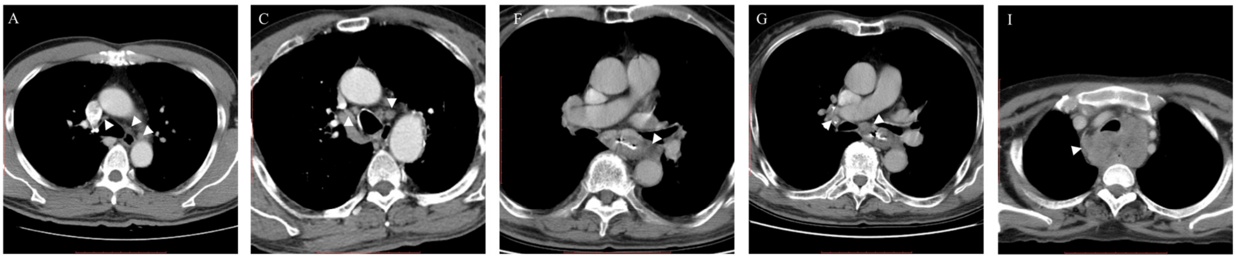

Central vein CT

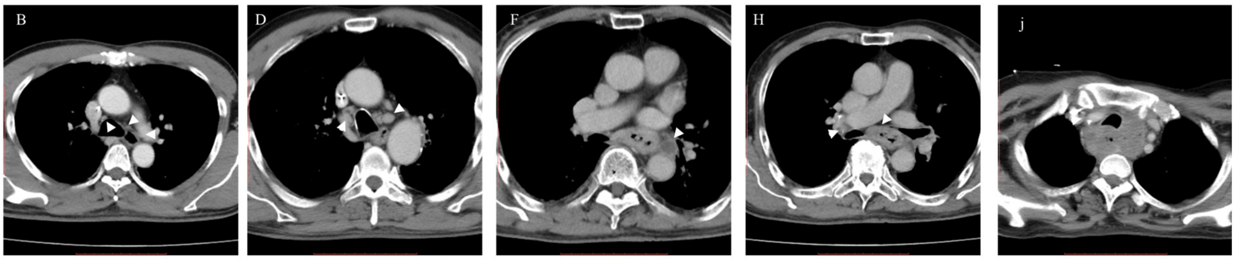

A (Peripheral vein CT)/ B (Central vein CT)	Small lymph node	Case number : 6
C(Peripheral vein CT)/ D (Central vein CT)	Cluster of lymph node	Case number : 1
E (Peripheral vein CT)/ F (Central vein CT)	Nectoric lymph node	Case number : 1
G (Peripheral vein CT)/ H (Central vein CT)	Enlarged LN	Case number : 2
I (Peripheral vein CT)/ J (Central vein CT)	Lymph node close to tumor	Case number: 2

Figure S5. Differences in N stage between peripheral vein and central vein CT and the reasons for N stage revision.

A/B: Small size lymph node and thoracic inlet
C/D: Cluster of lymph nodes
E/F: Necrotic lymph node
G/H: Enlarged lymph node
I/J: Lymph node close to tumor

Table S5. Stage migration after stage revision by central vein CT (pre-revision)

Stage	Clinical stage							
	TNM			CT (peripheral) +PET	TNM			CT (central) +PET
Case 1	T3	N2	M0	3	T3	N2	M0	3
Case 2	T2	N3	M0	4a	T2	N3	M0	4a
Case 3	T3	N2	M0	3	T3	N2	M0	3
Case 4	T2	N2	M0	3	T2	N2	M0	3
Case 5	T3	N2	M0	3	T3	N2	M0	3
Case 6	T3	N3	M0	4a	T3	N3	M0	4a
Case 7	T3	N3	M0	4a	T2	N1	M0	2
Case 8	T3	N2	M0	3	T4	N2	M0	4a
Case 9	T4	N2	M0	4a	T3	N2	M0	3
Case 10	T3	N0	M0	2	T3	N0	M0	2
Case 11	T2	N1	M0	2	T2	N1	M0	2
Case 12	T3	N2	M0	3	T3	N2	M0	3
Case 13	T3	N1	M0	3	T3	N3	M0	4a
Case 14	T3	N2	M0	3	T3	N2	M0	3
Case 15	T3	N1	M0	3	T3	N1	M0	3
Case 16	T4	N2	M0	4a	T3	N2	M0	3
Case 17	T3	N0	M0	2	T3	N0	M0	2
Case 18	T3	N2	M0	3	T3	N1	M0	3
Case 19	T2	N1	M0	2	T3	N2	M0	3
Case 20	T3	N2	M0	3	T3	N0	M0	2
Case 21	T3	N2	M0	3	T3	N2	M0	3
Case 22	T3	N2	M0	3	T3	N2	M0	3
Case 23	T3	N1	M0	3	T3	N3	M0	4a
Case 24	T3	N3	M0	4a	T3	N3	M0	4a
Case 25	T3	N2	M0	3	T3	N3	M0	4a
Case 26	T3	N1	M0	3	T3	N1	M0	3
Case 27	T3	N2	M0	3	T4	N2	M0	4a
Case 28	T3	N1	M0	3	T3	N1	M0	3
Case 29	T3	N2	M0	3	T3	N2	M0	3
Case 30	T4	N2	M0	4a	T4	N3	M0	4a
Case 31	T4	N0	M0	4a	T2	N0	M0	2
Case 32	T3	N1	M0	3	T3	N1	M0	3
Case 33	T4	N3	M0	4a	T4	N3	M0	4a
Case 34	T4	N2	M0	4a	T4	N2	M0	4a
Case 35	T3	N3	M0	4a	T3	N3	M0	4a
Case 36	T3	N0	M0	2	T3	N1	M0	3
Case 37	T4	N2	M0	4a	T4	N0	M0	4a
Case 38	T3	N1	M0	3	T3	N1	M0	3
Case 39	T2	N2	M0	3	T3	N2	M0	3
Case 40	T2	N2	M0	3	T3	N2	M0	3
Case 41	T3	N0	M0	2	T3	N0	M0	2
Case 42	T2	N3	M0	4a	T4	N3	M0	4a
Case 43	T3	N2	M0	3	T3	N1	M0	3
Case 44	T3	N0	M0	2	T3	N2	M0	3
Case 45	T3	N3	M0	4a	T4	N3	M0	4a
Case 46	T4	N1	M0	4a	T4	N1	M0	4a
Case 47	T3	N1	M0	3	T4	N2	M0	4a
Case 48	T4	N2	M0	4a	T3	N2	M0	3
Case 49	T4	N1	M0	4a	T4	N1	M0	4a
Case 50	T3	N1	M0	3	T4	N2	M0	4a

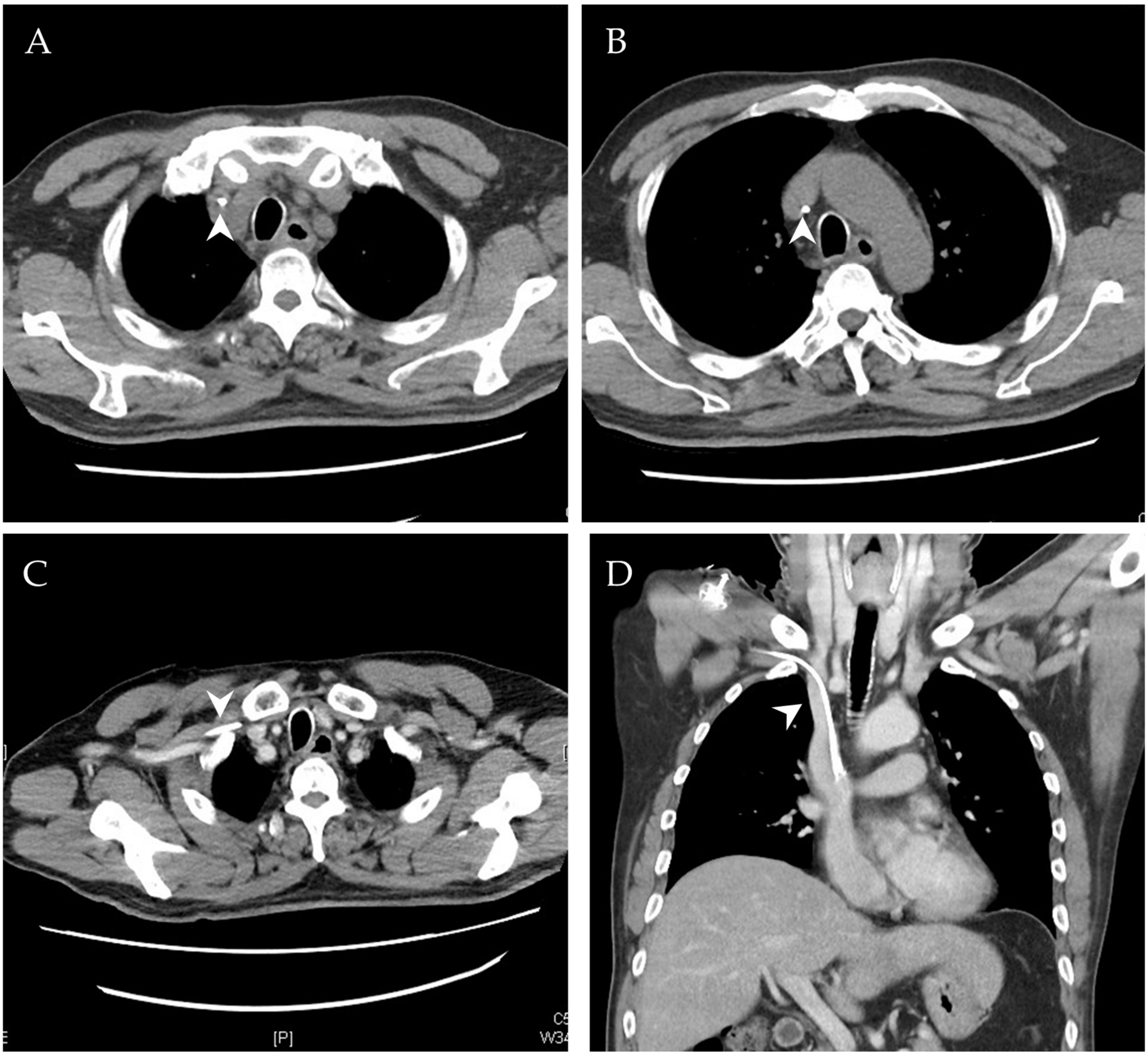

Figure S6. Actual catheter image in non-enhanced and central vein enhanced CT.

A. Catheter image (white arrow) in non-enhanced CT. (upper SVC)
B. Catheter image (white arrow) in non-enhanced CT. (junction site between left brachiocephalic vein and SVC)
C. Catheter image (white arrow) in enhanced CT via central vein enhancement. (cross section)
D. Catheter image (white arrow)in enhanced CT via central vein enhancement. (sagittal section) Smooth venous pattern was revealed and no more peripheral vein regurgitation was noted.
